# Supplementary material for: An Integrative Analysis of Transcriptome, Proteome and Hormones Reveals Key Differentially Expressed Genes and Metabolic Pathways Involved in Flower Development in Loquat
Source: Int J Mol Sci. 2020 Jul 20;21(14):5107. doi: 10.3390/ijms21145107 (PMC7404296; doi:10.3390/ijms21145107)
Supplement: Supplementary file 1 [file ijms-21-05107-s001.zip › Supplementary Figures.docx]

Supplementary Figures


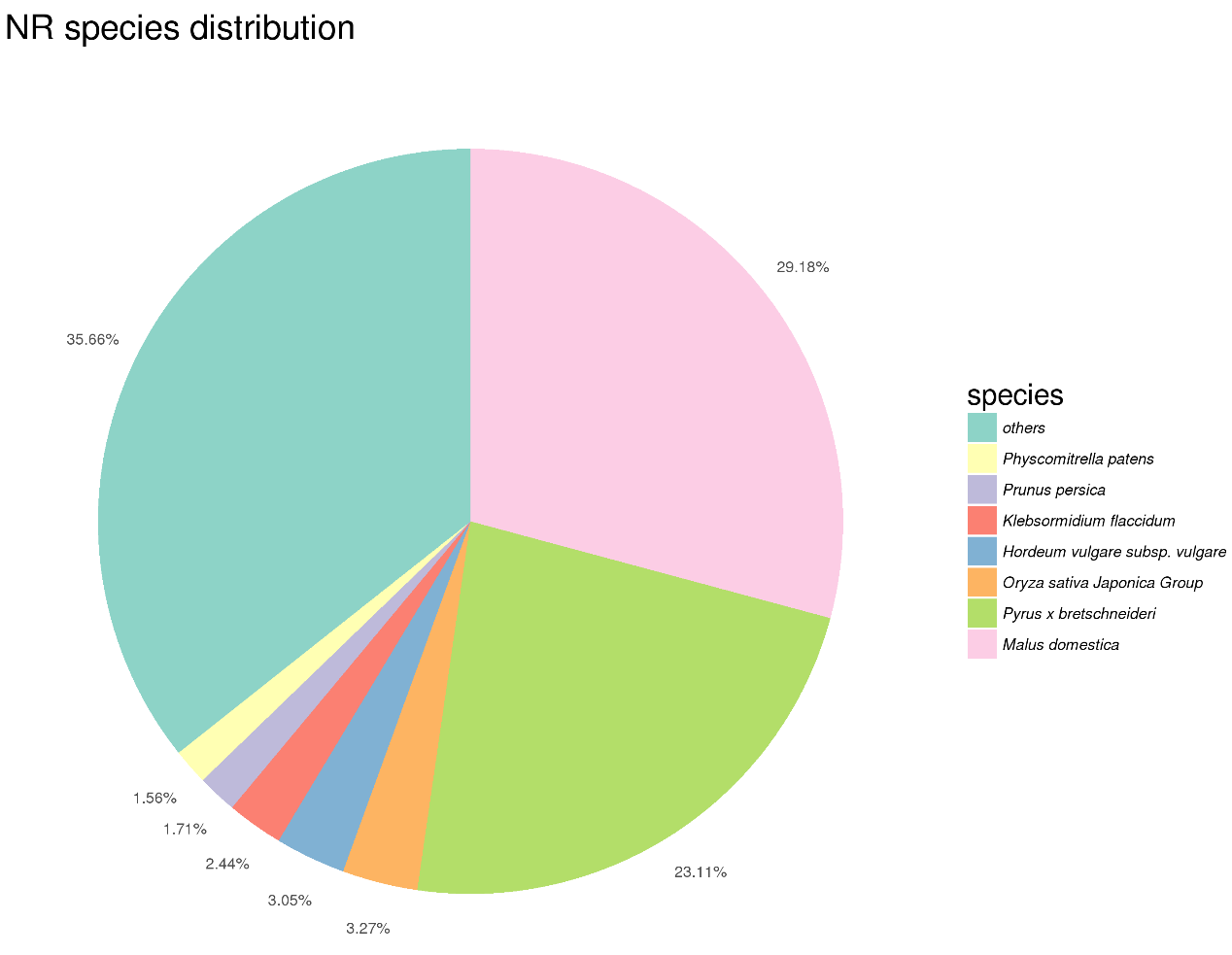


Figure S1. Species distribution of the top BLAST hits for all homologous sequences.


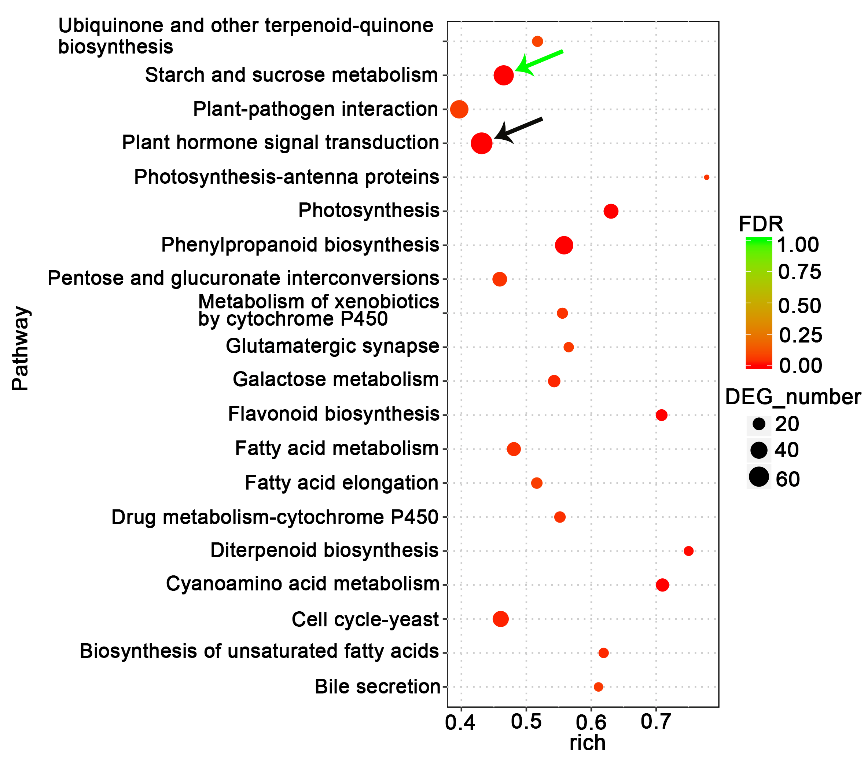


Figure S2. The enrichment analysis of DEGs for FA vs FBD.


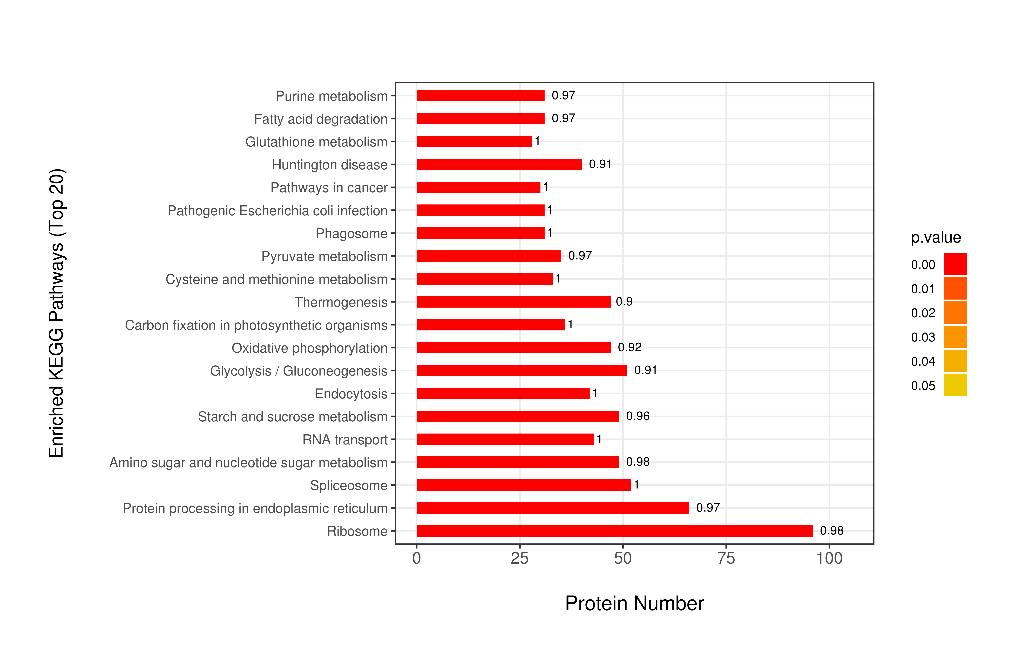


Figure S3. KEGG pathway enrichment analysis of the identified proteins.


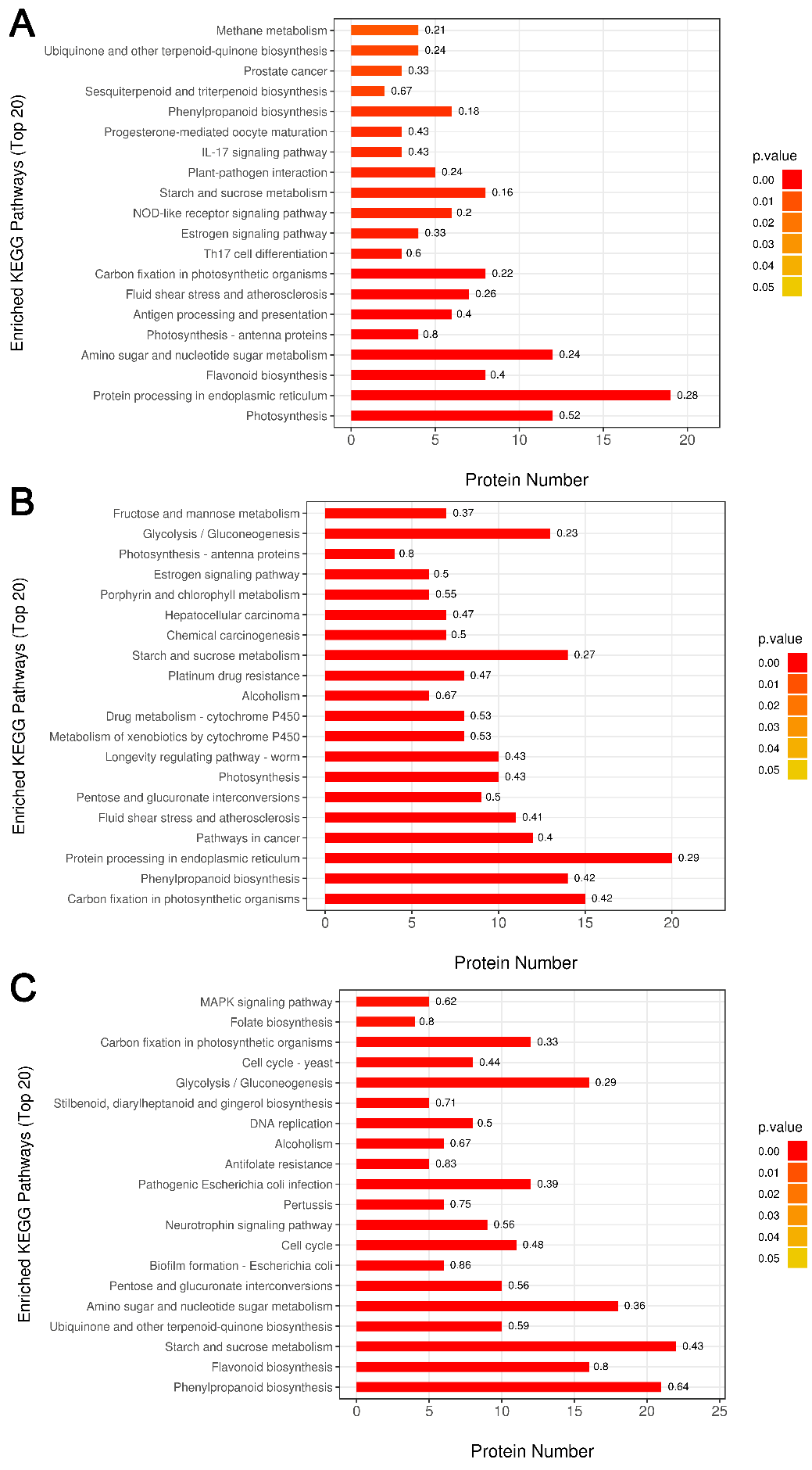


Figure S4. KEGG pathway enrichment analysis of DAPs of FBE vs FBD, FA vs FBE, FA vs FBD.
