# Supplementary material for: An Integrative Analysis of Transcriptome, Proteome and Hormones Reveals Key Differentially Expressed Genes and Metabolic Pathways Involved in Flower Development in Loquat
Source: Int J Mol Sci. 2020 Jul 20;21(14):5107. doi: 10.3390/ijms21145107 (PMC7404296; doi:10.3390/ijms21145107)
Supplement: Supplementary file 1 [file ijms-21-05107-s001.zip › Supplementary Tables.docx]

Supplementary Tables

Table S1 The RNA sequencing quality of floral bud development in loquat

| Sample | Total nucleotide (nt) | Clean Reads No. | Clean Reads (%) | Q30 (%) | Low-quality Reads Rate(%) |
| --- | --- | --- | --- | --- | --- |
| FBD-1 | 7,066,725,900 | 45,739,476 | 97.09 | 93.49 | 0.84 |
| FBD-2 | 6,943,122,000 | 45,292,314 | 97.85 | 92.45 | 1.33 |
| FBD-3 | 7,352,386,800 | 47,234,952 | 96.37 | 92.87 | 1.01 |
| FBE-1 | 7,062,645,000 | 45,486,924 | 96.61 | 93.61 | 0.86 |
| FBE-2 | 7,385,999,400 | 47,673,962 | 96.82 | 92.74 | 0.98 |
| FBE-3 | 7,380,462,300 | 47,775,636 | 97.1 | 93.19 | 0.91 |
| FA-1 | 7,087,296,900 | 45,533,950 | 96.37 | 92.82 | 0.96 |
| FA-2 | 7,212,035,100 | 46,826,136 | 97.39 | 93.04 | 0.93 |
| FA-3 | 7,087,574,400 | 45,716,016 | 96.75 | 92.53 | 1.18 |

Table S2 Summary of *de novo* assembly for floral development

|  | Transcript | Unigene |
| --- | --- | --- |
| Total Length (bp) | 368,629,837 | 103,697,404 |
| Sequence Number | 348,512 | 150,219 |
| Mean Length (bp) | 1057.7 | 690.3 |
| N50 (bp) | 1849 | 1164 |
| N50 Sequence Number | 62,827 | 22,727 |
| N90 (bp) | 420 | 264 |
| N90 Sequence Number | 218,647 | 105,365 |
| GC % | 43.11 | 44.38 |

Table S3 Functional annotation for flower development

| Database | Number | Percentage (%) |
| --- | --- | --- |
| NR | 67,072 | 44.65 |
| GO | 26,102 | 17.38 |
| KEGG | 6714 | 4.47 |
| eggNOG | 58,246 | 38.77 |
| Swissprot | 57,557 | 38.32 |
| In all database | 3737 | 2.49 |

Table S4. KEGG pathway enrichment analysis of DEGs for FBE vs FBD

| **Pathway_ID** | **Pathway** | **DEG_number** | **pvalue** | **FDR** |
| --- | --- | --- | --- | --- |
| ko00941 | Flavonoid biosynthesis | 13 | 3.70665E-06 | 0.000712115 |
| ko00940 | Phenylpropanoid biosynthesis | 28 | 7.05064E-06 | 0.000712115 |
| ko00982 | Drug metabolism - cytochrome P450 | 13 | 5.13363E-05 | 0.003456643 |
| ko00904 | Diterpenoid biosynthesis | 9 | 8.42322E-05 | 0.004253726 |
| ko00980 | Metabolism of xenobiotics by cytochrome P450 | 12 | 0.000111019 | 0.004485147 |
| ko00073 | Cutin, suberine and wax biosynthesis | 9 | 0.000157033 | 0.005286784 |
| ko00480 | Glutathione metabolism | 18 | 0.00021756 | 0.006278151 |
| ko00350 | Tyrosine metabolism | 13 | 0.000921884 | 0.023277574 |
| ko04152 | AMPK signaling pathway | 18 | 0.00113614 | 0.025500031 |
| ko01210 | 2-Oxocarboxylic acid metabolism | 14 | 0.00178941 | 0.036146082 |
| ko00950 | Isoquinoline alkaloid biosynthesis | 8 | 0.002444832 | 0.044710758 |
| ko00020 | Citrate cycle (TCA cycle) | 14 | 0.002858169 | 0.044710758 |
| ko04111 | Cell cycle - yeast | 20 | 0.002877425 | 0.044710758 |
| ko01200 | Carbon metabolism | 37 | 0.01246489 | 0.179850556 |
| ko04914 | Progesterone-mediated oocyte maturation | 9 | 0.01369804 | 0.184466939 |
| ko04730 | Long-term depression | 4 | 0.01623316 | 0.204943645 |
| ko04110 | Cell cycle | 20 | 0.01802212 | 0.205226365 |
| ko00620 | Pyruvate metabolism | 16 | 0.01876275 | 0.205226365 |
| ko00630 | Glyoxylate and dicarboxylate metabolism | 13 | 0.01930347 | 0.205226365 |
| ko04075 | Plant hormone signal transduction | 33 | 0.02081621 | 0.210243721 |
| ko04390 | Hippo signaling pathway | 7 | 0.03168818 | 0.295346128 |
| ko03320 | PPAR signaling pathway | 8 | 0.03216641 | 0.295346128 |
| ko04113 | Meiosis - yeast | 14 | 0.04177831 | 0.366922549 |
| ko04530 | Tight junction | 6 | 0.04863779 | 0.409368066 |

Table S5. KEGG pathway enrichment analysis of DEGs for FA vs FBE

| **Pathway_ID** | **Pathway** | **DEG_number** | **pvalue** | **FDR** |
| --- | --- | --- | --- | --- |
| ko00940 | Phenylpropanoid biosynthesis | 49 | 1.42109E-11 | 3.36798E-09 |
| ko00195 | Photosynthesis | 30 | 1.50672E-09 | 1.78546E-07 |
| ko00941 | Flavonoid biosynthesis | 18 | 1.16119E-07 | 9.17339E-06 |
| ko00040 | Pentose and glucuronate interconversions | 32 | 6.69686E-07 | 3.96789E-05 |
| ko00500 | Starch and sucrose metabolism | 53 | 4.36495E-06 | 0.000206899 |
| ko00073 | Cutin, suberine and wax biosynthesis | 12 | 4.61431E-05 | 0.001822652 |
| ko00196 | Photosynthesis - antenna proteins | 8 | 6.10934E-05 | 0.002068446 |
| ko00460 | Cyanoamino acid metabolism | 16 | 0.000552762 | 0.016375574 |
| ko00052 | Galactose metabolism | 17 | 0.000918656 | 0.024191283 |
| ko04111 | Cell cycle - yeast | 30 | 0.001123745 | 0.026632757 |
| ko00380 | Tryptophan metabolism | 12 | 0.001471868 | 0.031712065 |
| ko04724 | Glutamatergic synapse | 12 | 0.002431214 | 0.048016477 |

Table S6. KEGG pathway enrichment analysis of DEGs for FA vs FBD

| **Pathway_ID** | **Pathway** | **DEG_number** | **pvalue** | **FDR** |
| --- | --- | --- | --- | --- |
| ko00940 | Phenylpropanoid biosynthesis | 48 | 8.57857E-08 | 2.11891E-05 |
| ko00195 | Photosynthesis | 29 | 1.05442E-06 | 9.08679E-05 |
| ko00460 | Cyanoamino acid metabolism | 22 | 1.10366E-06 | 9.08679E-05 |
| ko00500 | Starch and sucrose metabolism | 60 | 8.74999E-06 | 0.000540312 |
| ko00941 | Flavonoid biosynthesis | 17 | 2.0192E-05 | 0.000997484 |
| ko04075 | Plant hormone signal transduction | 72 | 3.08169E-05 | 0.001268627 |
| ko00904 | Diterpenoid biosynthesis | 12 | 0.000151581 | 0.005348658 |
| ko04111 | Cell cycle - yeast | 35 | 0.000798119 | 0.024641927 |
| ko00052 | Galactose metabolism | 19 | 0.001157626 | 0.031770402 |
| ko01040 | Biosynthesis of unsaturated fatty acids | 13 | 0.001425028 | 0.035198192 |
| ko01212 | Fatty acid metabolism | 25 | 0.002076755 | 0.046472144 |
| ko00982 | Drug metabolism - cytochrome P450 | 16 | 0.002257756 | 0.046472144 |
| ko00040 | Pentose and glucuronate interconversions | 28 | 0.00274071 | 0.049770641 |
| ko00980 | Metabolism of xenobiotics by cytochrome P450 | 15 | 0.002821008 | 0.049770641 |

Table S14. Primer sequences and annealing temperatures used for qRT-PCR.

| Gene | Primer sequences (5 to 3) | Annealing temperatures (°C) |
| --- | --- | --- |
| *Ejactin* | AATGGAACTGGAATGGTCAAGGC | 55 |
|  | TGCCAGATCTTCTCCATGTCATCCCA |  |
| *AP1* | CTCGGATGCTTTGCTGCTTG | 55 |
|  | AGTACGTTCAGGGTTGGTCC |  |
| *AP2* | GACGGTCGGAGGACCATCCAGC | 55 |
|  | CGATAAAACGTAACGCCCCGAT |  |
| *FT* | GTTGTTGGACGAGTGGTAG | 55 |
|  | TAACCTCTTTATTGCCGTAG |  |
| *GA20ox2* | AACCATGGCAAAACCTCCCT | 54 |
|  | GAGTGGGACTCGAAGCTCTG |  |
| *GAI* | AAGAGGGCCGTTGATTGAGG | 54 |
|  | ACCGTCCATTACAACCCGTC |  |
| *SVP* | GAAGTCCCTTGAAGCTGGCT | 56 |
|  | CTCTTCCGCCAATTGCATCG |  |
| *SPL4* | GGATGACTACTCGGATGAAG | 56 |
|  | CAACCAAATCAGCCTCAC |  |
| *AG* | AGCGGATCGAAAACACGAC | 54 |
|  | GCATCACAGAGCACAGAGAGT |  |
| *SOC1* | GCGTTATCAGAAGCATGCGAAAGA | 54 |
|  | GCTCGGACGTTGTAGACGCTC |  |
| *LFY* | GGATGACAACGACATGGACG | 54 |
|  | GGTGCTCCCTTTGTCTCTCT |  |
| *PI* | TCTGGGAAGATGGTTGAATAC | 55 |
|  | TGAGCTCTACTTGCATGCTGT |  |
| *G3OX1* | AAGAGATGAAGAGGCTTGCCT | 55 |
|  | ACTTGCAAGCCGTCAATGTTG |  |
